# The Effect of Milking Frequency, Breed, and Stage of Lactation on the Milk Fat Globule Size and Fatty Acid Composition in Sheep’s Milk

**DOI:** 10.3390/foods12132446

**Published:** 2023-06-22

**Authors:** Theofilos Massouras, Aggeliki-Alexandra Charmanta, Panagiota Koutsouli, Maria Masoura, Ioannis Politis, Kasper Hettinga

**Affiliations:** 1 Laboratory of Dairy Research, Department of Food Science and Human Nutrition, Agricultural University of Athens, Iera Odos 75, 11855 Athens, Greece; 2 Laboratory of Animal Breeding and Husbandry, Department of Animal Science, Agricultural University of Athens, Iera Odos 75, 11855 Athens, Greece; panagiota@aua.gr (P.K.); i.politis@aua.gr (I.P.); 3 School of Chemical Engineering, University of Birmingham, Birmingham B12 2TT, UK; m.masoura@bham.co.uk; 4 Dairy Science and Technology Group, Food Quality and Design, Wageningen University, 6708 WG Wageningen, The Netherlands; kasper.hettinga@wur.nl

**Keywords:** sheep milk, milking frequency, fat globule, fatty acid composition

## Abstract

This study examined the effects of milking frequency, breed, and stage of lactation on the milk fat globules (MFG) size and fatty acids (FA) composition of sheep milk. Milk from Karagouniko (*n* = 13) and Chios (*n* = 13) ewes was sampled postpartum on the 93rd, 101st, 108th, 121st, 156th, and 188th days of lactation. On the 108th day, the ewes were divided randomly into two milking groups: Once daily at 06:00 a.m. or twice daily at 06:00 a.m. and 16:00 p.m. Morphometric characteristics of MFG and FA composition were determined for each sample. Once versus twice daily milking had no effect on MFG dimensions, which tended to vary according to breed (smaller MFG were secreted from Chios with *p* = 0.065), while the stage of lactation had a significant effect (*p* < 0.001). FA composition differed significantly according to the stage of lactation and breed. The FA profile of the Karagouniko breed showed higher concentrations of short-chain FA. The milk samples from late lactation were characterized by higher concentrations of mono-unsaturated FA (MUFA) compared to early and mid-lactation. Moreover, correlations were found between the average diameter of MFG and FA concentrations, where the size of MFG was positively correlated with saturated FA (SFA) and negatively correlated with MUFA.

## 1. Introduction

From a global perspective, sheep milk and its corresponding products are less consumed than cow milk and its products, although in Mediterranean countries, sheep milk is widely known with a rapidly growing consumption. These countries produce approximately 5.15 million tonnes, representing 44% of the world’s sheep milk production, according to Food and Agriculture Organization of the United Nations. Statistical Database FAOSTAT [1]. The Mediterranean region provides diverse environments (high altitude regions, coastal, wetlands, and arid regions) and variable pasture conditions where well-adapted local sheep breeds are reared [2,3]. Most sheep milk is sold to industries and processed into traditional cheese types, many of which are Protected Denomination of Origin (PDO) cheeses for gourmet and export markets (e.g., Feta, Pecorino Romano, Manchego, Sierra da Estrela, Fiore Sardo, Roquefort) [4,5]. 

Milk fat plays a significant role in the properties of milk and its nutritional value. It contains approximately 400 different fatty acids (FA), which makes it the most complex of all natural fats. The milk FA are derived half from the feed (incl. rumen), and the other half comes from de novo synthesis in the mammary gland [6]. Milk FA (≤C15:0 and a portion of C16:0) are synthesized de novo in the mammary gland from acetate, propionate, and β-hydroxybutyrate, whereas a portion of C16:0 and all ≥C17:0 originate from dietary lipids [7,8,9,10].

Milk fat is secreted as droplets called milk fat globules (MFG) by a unique secretion process in the mammary gland. The diameter of the MFG varies between 0.2 and 15 μm, and its composition is observed to vary by size, adding more complexity to the study of its structure and function. The core of MFG is composed of triacylglycerols, enveloped by the milk fat globule membrane (MFGM), which mainly consists of phospholipids, proteins, enzymes, and cholesterol [11,12,13,14,15]. MFGM components are characterized as beneficial for health [16,17] due to the protective effect of MFGM against infectious diseases, which is in part through the modulation of the intestinal immune response and the gut microbiota [18,19,20]). MFG show a wide range of sizes depending on many genetic and physiological factors, such as species, genotype, stage of lactation, season, age of ewe, and feeding. A wide range of MFG sizes is observed, even in animals of the same breed [9,12,13,14,15,16]. In general, the diameter of MFG is considered a factor that influences the differences in fatty acid composition, since the fatty acid composition differs between the membrane and the core. It is reported that the average diameter of sheep’s MFG is between 2.8 and 4.9 μm, while the range of average diameters in cows varies between 3.5 and 5.5 μm and in goats between 2.2 and 2.8 μm [11]. The various dimensions of the secreted MFG are linked to technological and organoleptic characteristics of milk [21], but also to its nutritional value [22,23].

Many factors are associated with the variations in the amount and fatty acid composition of milk lipids. They may be of animal origin, i.e., related to genetics (breed and selection), stage of lactation, and ruminal fermentation, or they may be feed-related factors, i.e., related to fiber and energy intake, dietary fats, and seasonal and regional effects. The four most abundant FA in the milk of ruminants are C16:0 with cis-9 C18:1 with C14:0 with C18:0, accounting for more than 60% of the total. Variations in FA composition exist among dairy cows, sheep, and goats [17,24,25]. Regarding the saturated FA in sheep milk, about 17% are short-chain FA (C4:0; C6:0; C8:0; C10:0), in contrast to cow milk, which contains only 11%. About 24–29% of FA of sheep’s milk fat is mono-unsaturated FA (MUFA). poly-unsaturated FA (PUFA) reaches 4% of FA content, with the most important being the linoleic and linolenic acids. The content of Conjugated linoleic acid (CLA) (1.2%) in sheep milk exhibits a greater amount compared with other ruminant milk, 0.7 and 0.6 for cow and goat milk, respectively [17,24]. Specifically, research in the field of FA in sheep’s milk composition indicates that short-chain fatty acids, caproic (C6:0), caprylic (C8:0), and capric (C10:0) acids are present in greater amounts than in cow’s milk and are linked with better digestibility of sheep milk lipids [17,24]. 

The size of the MFG is tightly associated with its composition, with a higher content of mono- and poly-unsaturated fatty acids and phospholipids in small vs. large MFG [15]. Therefore, small MFG are associated with beneficial effects on human health, considering that they contain more phospholipids and bioactive proteins and are better digestible due to their smaller size [11,16,17]. Many authors have stated that poly-unsaturated fatty acids (PUFA), such as n-3 FA and CLA, are associated with the membrane of MFG. Oppositely, the core is richer in saturated FA, such as C16:0, C14:0, but also n-6 FA. Thus, the ratio of unsaturated to saturated FA is lower in the fat globule core than in the membrane [26]. Milk fat composition and MFG dimensions are influenced by the stage of lactation [13,27,28]. Furthermore, milking frequency is a procedure that stimulates mammary function and regulates the yield and composition of milk [29,30,31], which may thereby indirectly affect the size of MFG [27,32,33] in cows, ewes, and goats. The above variations among dairy species rely on the cisternal storage capacity of animals [29,34]. These observations highlight the fact that the secretion mechanism of milk fat is different among the species, and the interaction between composition and structure of MFG shows wide variation. Although modification of FA profile through milking frequency was previously observed for bovine milk [35], data are lacking, and no accurate correlation between the milking frequency and the morphometric characteristics of MFG in sheep’s milk exist. 

Studies on the morphometric characteristics of MFG are limited, mainly because various confounders indirectly affect MFG size. In this context, our research goal is focused on finding the correlation of MFG size with fatty acid composition and how they are affected by the lactation stage and milking frequency treatment. Thus, the objectives of this work were: (a) To study the variation of the MFG size and (b) to investigate how the factors: milking frequency, breed, and stage of lactation affect the morphometric characteristics of MFG and the FA composition. These effects on the size of MFG and FA composition during lactation could provide an opportunity to regulate milk properties to achieve better dairy product quality through the improvement of its nutritional profile.

## 2. Materials and Methods

### 2.1. Animals and Sample Collection

This study was performed with Greek sheep, 13 Chios and 13 Karagouniko multiparous ewes, reared at the Animal Farm Station of the Agricultural University of Athens (AUA) under the same diet (Table S1) and handling conditions in a sheepfold with a free-stall housing system. The selected breeds are of great importance in the production of sheep milk in the country. Karagouniko breed is reared under semi-mountainous or lowland regions of Central Greece under extensive or semi-extensive management regimes [36] with a moderate milk yield. Chios is considered a highly productive and prolific dairy breed mostly distributed in Northern Greece, usually reared in semi-intensive or intensive housing conditions [37]. At the beginning of the experiment, all ewes were healthy and did not show signs of metabolic diseases or other diseases (e.g., lameness). The estrous cycle of the ewes was synchronized with progesterone sponges (Ovigest, Laboratories Hipra S.A., Amer, Girona, Spain). The mating was natural, and lambing (one or two lambs per ewe) occurred in December (7–21 December). The weaning of the lambs was 42 days postpartum and after this day, the ewes were milked twice daily (at 06:00 a.m. and at 16:00 p.m.) in 1 × 12 units of a milking parlor (Westfalia), applying a pulsation ratio of 50:50 and pulsation rate of 150 cycles min^−1^ with 37.5 kPa vacuum level. An interval of 12 h was left between milkings. During the preliminary period, milk samples were received postpartum on day 93rd and 101st (d). On the 108th day postpartum, the ewes were divided randomly into two treatment groups: Once daily milking (at 06:00 a.m., *n* = 14) and twice daily milking (at 06:00 a.m. and 16:00 p.m., *n* = 12) and milk samples were collected on the 108th, 121st, 156th, and 188th days of lactation stage, between March and June (Figure S1). Milk yields were recorded, and the chemical composition of milk samples was determined for protein, fat, lactose, and total solids using MilkoScan FT120 (Foss Electric, Hillerod, Denmark). The experiment was carried out in accordance with the national legislation and the guidelines of the Research Ethics Committee of the Agricultural University of Athens regarding the protection and welfare of animals used for experimental and other scientific purposes. 

### 2.2. Morphometric Analysis of Milk Fat Globules

The milk fat globule size distribution was determined by laser light diffraction using a Mastersizer 2000 (Malvern Instruments Ltd., Malvern, UK). The experiments were performed at room temperature. Milk samples were diluted (1:1) with 35 mmol/L EDTA/NaOH pH 7.0 prior to measurements to dissociate casein micelles. The operating procedure requires a small quantity of each sample to be added to the tank (reservoir of 100 mL millipore water) until the obscuration reaches 10%. The introduced amount of milk sample fluctuated from 50 to 100 μL, depending on the size and amount of milk fat globules. Finer particles cause greater obscuration, and therefore, a smaller amount was needed. The sample was diluted into the dispersant liquid under moderate stirring. The suspension reached the measurement cell through the pump that was operating at 800 rpm. The refractive index of the fat globule and the dispersant (water) was 1.45 and 1.33, respectively. The absorption of fat globules was taken as 0.001. We have checked that different obscuration levels (2%, 4–5%, and 15%) did not significantly influence the measured particle size distribution, 10% obscuration was chosen as the optimal condition for fat particles. Standard parameters were calculated by the Malvern Panalytical Software (Malvern Instruments Ltd., Malvern, UK) [38]: The surface weighted mean diameter D_(3,2)_ defined as Σn_i_d_i_^3^/Σn_i_d_i_^2^, the mean volume weighted diameter D_(4, 3)]_ defined as Σn_i_d_i_^4^/Σn_i_d_i_^3^ (where n_i_ is the number of fat globules in a size class of diameter d_i_), the volume median diameter d_(v, 0.5)_ which corresponds to the size that 50% of all particles are smaller and 50% are larger, d_(v, 0.9)_ and d_(v, 0.1)_ represent respectively 90% and 10% of all particles that have a diameter smaller than this value. The value of the size distribution width, defined as Span = d_(v, 0.9)_ − d_(v, 0.1)_/d_(v, 0.5)_, reduces as the distribution becomes narrower. The software presents these standard parameters by averaging the various dimensions of particles [39,40]. The shape of the curve of particle size distribution, plotted using a logarithmic size scale, is defined by measuring the volumes of particles at 100 different sizes.

### 2.3. Fatty Acid Analysis

Freeze-dried milk samples were directly trans-esterified to fatty acid methyl esters (FAMEs) using the method of Massouras et al. [41] with slight modifications. The amount of 120–160 mg of each dry sample was methylated by 2 mL of freshly prepared 0.5 M KOH in CH_3_OH solution at 50 °C for 30 min, followed by 2 mL of boron trifluoride-methanol solution (BF_3_) for 30 min at 50 °C. Hexane (3 mL, HPLC grade ≥ 98%) was added to the reaction mixture and allowed to react for 10 min at ambient temperature. The hexane solution allowed the recovery of FAMEs, and 1 mL of the upper hexane layer was transferred into a GC vial. The composition of the fatty acids was determined by gas chromatography using a Shimadzu gas chromatograph (model GC-17A, Columbia, MD, USA) with a Shimadzu GC-2014 GC AOC-20i auto-injector, equipped with a flame ionization detector (FID). Separation of fatty acid methyl esters was achieved on an SP-2560 capillary column (75 m × 0.18 mm I.D., 0.14 μm; Supelco Inc., Bellefonte, PA, USA). The flow rate of carrier gas (helium) was 1 mL·min^−1^, the injector temperature was 250 °C, and the detector temperature was 270 °C. The injection volume was 1 μL (split 1:40). The temperature program was as follows: The initial temperature was held at 75 °C for 5 min after injection and then programmed to increase at 5 °C/min to 150 °C, to hold for 5 min, then to increase to 220 °C at 7 °C/min and hold for 20 min. Fatty acid peaks were recorded and integrated using Shimadzu GC solution software (Shimadzu Corporation, Kyoto, Japan). Individual fatty acids were identified by comparing their retention times with known fatty acid methyl ester standards (Supelco 37 Component FAME Mix, purchased from Sigma-Aldrich, Taufkirchen, Germany). The individual FA content was expressed as a percentage of the total FA detected as FAMEs. SFA, PUFA, MUFA, ω6 and ω3 FA were calculated as the sum of the percent content of all saturated, poly-unsaturated, mono-unsaturated, ω6 and ω3 FA, respectively. PUFA/SFA and ω6/ω3 ratios were calculated by dividing PUFA by SFA and ω6 by ω3, respectively. The atherogenicity (AI) and thrombogenicity (TI) indices were calculated with the following formulas [42].

(1) AI=[C12:0+(4 × C14:0)+C16:0]/[∑MUFA+∑(ω3)PUFA+∑(ω6)PUFA]

(2) TI=(C14:0+C16:0+C18:0)/[0.5×∑MUFA+0.5×∑(ω6)PUFA+3×∑(ω3)PUFA+(ω3/ω6)]

### 2.4. Statistical Analysis

For data analysis, we used the SPSS 21.0 statistical package [43]. All data were analyzed according to MFG size parameters and FA composition. The variables considered for fat globules size were: The surface-weighted mean diameter D_(3,2)_, the volume-weighted mean diameter D_(4, 3)_, and the volume median diameter d_(v, 0.5)_, d_(v, 0.9),_ and d_(v, 0.1)_. For the statistical analysis of FA composition, the percentages of individual FAMEs, also the sums of SFA, MUFA, and PUFA, were treated as dependent variables. The values of iso-C13:0, anteiso-C13:0, iso-C15:0, C18:1t16, C18:3n6, C18:3, C20:2n6, C20:3n9, C20:3n6 fatty acids did not meet the ANOVA assumptions (normal distribution and homogeneity of variances within fixed factor classes), as they were found at negligible amounts in a few milk samples. For this reason, they were not included in the statistical analysis, and results are not shown for them. During the preliminary period, the data analysis considered the fixed effect of breed at two points of lactation (93rd and 101st days postpartum). During the experimental period, the analysis was carried out with a repeated measures analysis of variance (GLM) concerning the fixed effects of breed and milking frequency treatment at four points of lactation (108th, 121st, 156th, 188th days postpartum). The repeated fixed factor was time (four samplings during the lactation stage). The ewe was considered as a random factor nested within breed. The model used was:

Y_ijk_ = μ + B_i_ + T_j_ + L_k_ + B_i_ × T_j_ + B_i_ x L_k_ + T_j_ x L_k_ + B_i_ × T_j_ × L_k_ + e_ijk_ (3)

where:
μ = the mean
B_i_ = the fixed effect of breed with i = 1, 2 (1: Karagouniko, 2: Chios)
T_j_ = the fixed effect of milking frequency with j = 1, 2 (1: One milking per day, 2: Two milkings per day)
L_k_ = the repeated fixed effect of lactation stage with k = 1 to 4 representing: 1 = 108th d after birth, 2 = 121st d, 3 = 156th d and 4 = 188th d.
e_ijk_ = the random error assumed to be normally and independently distributed with zero expectation and common variance σ^2^.

Additionally, linear simple correlations were calculated between the FA variables and average volume weighted diameter D_(4,3)_. The limit of statistical significance was at *p* ≤ 0.05, and high significance was at *p* ≤ 0.001.

## 3. Results and Discussion

Table 1, Table 2 and Table 3 present the main effects of the fixed factors according to the preliminary and experimental periods, as no significant interactions were found between fixed factors. Table 4 presents the relationships between the MFG size and FA composition throughout lactation.

### 3.1. Fatty Acid Composition

Gas chromatography (GC-FID) analysis revealed the presence of 46 fatty acids, from C4:0 to C23:0. The values listed in the tables represent the percentage of the analyzed fatty acids, in accordance with the effect of milking frequency (Table 1), breed (Table 2), and lactation stage (Table 3). Fatty acids detected with a percentage below 0.1% were classified to a group of non-identified fatty acids (NIFA), as these could not be matched to the retention times of standard methyl esters. Saturated fatty acids (SFA) accounted for 63–68% of the total. Among the mono-unsaturated fatty acids (MUFA), which reached a total content of 25–27%, high proportions of oleic acid (C18:1n9) were identified. Regarding poly-unsaturated fatty acids (PUFA), the main identified fatty acids were linoleic (C18:2n6) and rumenic (C18:2cis-9trans-11) acids. In contrast with previous studies, a high amount of PUFA was found in all milk samples, ranging up to 8% [17,24,44]. Higher concentrations of PUFA could be attributed to the chemical composition of feed since PUFA are preformed dietary FA, which are transported via the blood to the mammary gland [45,46]. Fatty acid groups in all samples decreased in the order: SFA > MUFA > PUFA. For all milk samples, the major fatty acids were myristic (C14:0), palmitic (C16:0), stearic (C18:0), oleic (C18:1n9), and linoleic acid (C18:2n6), accounting for 10, 30, 12, 22, and 4% respectively. The concentrations of the above FA are in agreement with other studies [17,24].

### 3.2. Effect of Milking Frequency on Size of Milk Fat Globules and Fatty Acids’ Profile

**Figure 1:**
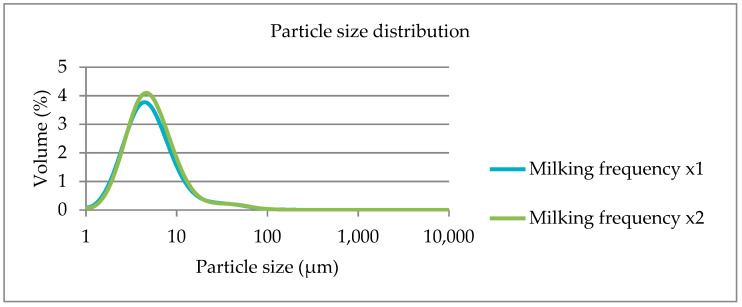
Fat globule size distribution in milk obtained from ewes treated with once (×1) and twice (×2) daily milking during the experimental period.

During the experimental period, the main effects of milking frequency, being once (×1) and twice (×2) daily milking, daily milk yield, daily fat yield and fat contents, MFG size parameters and FA composition of raw milk samples are presented in Table 1. The morphometric traits of MFG were not affected by the reduction of milking frequency. As shown in Figure 1, the fat globule size distributions were almost similar between milk samples obtained from once or twice daily milking.

A previous study on goat milk showed that milking intervals between 12 and 24 h did not affect the size of MFG [47]. Similarly, milking frequency had no effect on FA profile. In Table 1, all the FA values did not differ between milking frequencies, apart from C18:2trans-6 and C18:2trans-8, cis-13 that showed a significant reduction (*p* < 0.05) for the twice-daily milking treatment. The ewes milked once daily produced less milk. However, this difference was not significant (*p* = 0.138). Similarly, fat yield and fat content between milking once and twice daily did not differ. This observation was expected since no significant changes were observed in milk yield. Previous studies that investigated the effect of milking frequency reduction on milk yield and composition of dairy animals reported that once-daily milking caused a decrease in milk yield but increased the fat content [32,34,48]. The losses of milk production are provoked by an increase of the intramammary pressure due to the accumulation of milk in the udder, but also by the accumulation of feedback inhibitor lactation protein, which is synthesized by the mammary gland. The high fat content is a consequence of the concentration of milk components when milk yields are reduced [27,32,34]. Among different species and breeds, milk production shows variation that depends on the cisternal capacity of each animal. The above results indicated that the milking frequency reduction did not affect the lipid phase of sheep’s milk. There were no significant differences, neither in FA composition nor in size traits of MFG, between once and twice daily milking. Future studies should investigate the implications of shorter milking intervals on MFG traits in sheep milk.

### 3.3. Effect of Breed on Size of Milk Fat Globules and Fatty Acid Profile

**Figure 2:**
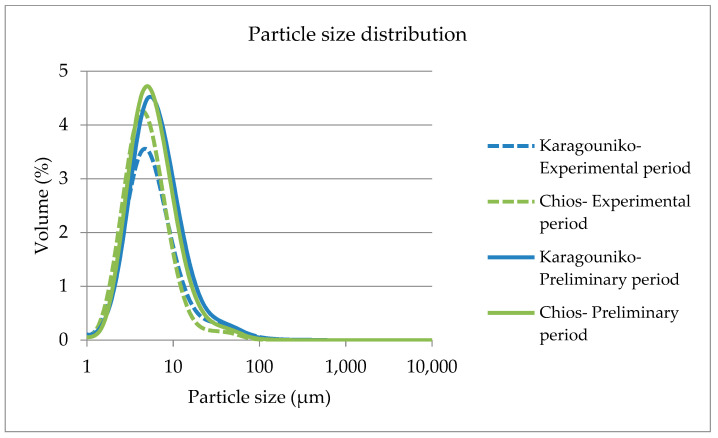
Fat globule size distribution in sheep milk of Karagouniko and Chios breed.

The range of values for MFG size distributions was reduced during the experimental period compared with the values of the preliminary period (Figure 2). The mean values of daily milk and fat yield and fat content, MFG size traits, and mean values of FA of Karagouniko and Chios sheep are shown in Table 2. The daily milk and fat yield were significantly different between breeds (*p* ≤ 0.05).

MFG size of Karagouniko breed was larger than that of Chios but did not differ significantly between the preliminary and experimental periods, with only one exception. The dimension of d_(0.5)_ was higher in Chios during the experimental period. Daily milk fat yield was higher in the Chios breed, in agreement with the significantly higher amounts of their daily milk production during both the preliminary and experimental periods. This observation confirms previous studies, which have reported that Chios ewes have higher milk yields than Karagouniko ewes [44]. The milk fat content of Karagouniko was numerically higher than Chios but not significantly different (*p* = 0.220). It is suggested that the larger MFG observed in Karagouniko milk may be a consequence of its slightly higher fat content. Previous research [49] stated that when cows produce high levels of fat, the synthesis of MFGM is a limiting factor since fat secretion involves a loss of resources. However, another study in sheep’s milk [50] indicates that fat secretion (g/mL) in milk was positively related with small MFG. Regarding the FA profile differences in relation to the breeds, our findings highlighted that the short-chain fatty acids (SCFA) were significantly different (*p* ≤ 0.05). Throughout lactation, the milk of Karagouniko ewes had higher content of butyric (C4:0) and caprylic acid (C8:0). Moreover, the content of caproic (C6:0), capric (C10:0), and lauric acid (C12:0) was higher for Karagouniko milk at all lactation stages, but the differences were significant only during the experimental period, where ewes had different milking frequencies. The milk of Chios ewe contained more palmitic acid (C16:0) than Karagouniko, both for the preliminary and experimental period (*p* ≤ 0.05). The percentages of total SFA, MUFA, and PUFA did not differ between the breeds.

The above findings demonstrate quantitative variability concerning the SCFA proportions between the two breeds. Since SCFA are linked with the characteristic flavor of sheep milk cheeses, but also with better digestibility [17], the milk from Karagouniko could be considered better for producing dairy products than Chios milk. Summarizing our findings, the milk of Karagouniko ewes contained more SCFA acids and was characterized by the secretion of larger MFG, while the smaller mean diameter of MFG in Chios milk showed a higher percentage of palmitic acid. The above findings agree with several studies that reported smaller amounts of short-chain fatty acids in small MFG [50,51,52]. Higher concentrations of palmitic acid (C16:0) in small fat globules were observed in the study of Martini et al. [50], whereas another study [17] reported a positive correlation between palmitic acid and large fat globules. Moreover, the increased secretion of small MFG from animals that produce higher milk yields, like Chios ewes in this study, has also been observed in high-yielding dairy cows, like Holsteins, where their mammary metabolic activity is high, resulting in elevated production of membrane material [53]. These changes in FA composition suggest that breed selection could be an important factor that contributes to the modification of milk properties.

### 3.4. Effect of Lactation Stage on Size of Milk Fat Globules and Fatty Acid Profile

**Figure 3:**
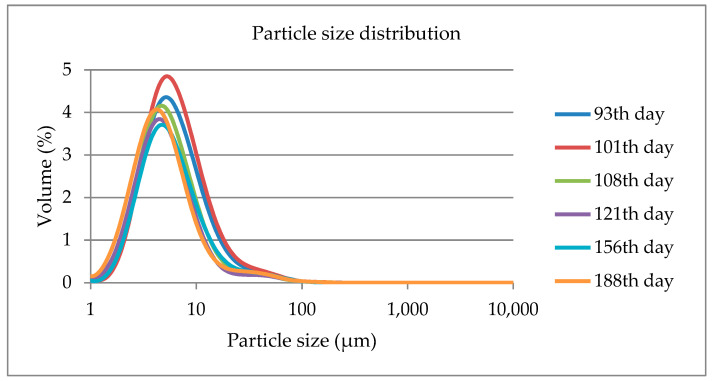
Fat globule size distribution in sheep’s milk throughout lactation.

The preliminary period coincided with the end of early and the onset of mid-lactation stage, while the experimental period showed the effects during the main part of mid and late lactation. No significant differences were found in the size dimensions of MFG during lactation (Figure 3 and Table 3). However, it can be observed visually from Figure 3 that the average diameter of MFG decreased at later lactation stages.

The fat globule size distribution in sheep milk during the whole lactation period showed the highest values on the 101st day. The fat globule size distributions were characterized by a greater width for early lactation stages (93rd and 101st d) compared to later lactation stages, which indicates greater secretion of small MFG as lactation progresses. Specifically, at the early lactation stage (preliminary period), the mean size of D_(4,3)_ was 4.3 μm (sd = ±1.6), whilst, at later lactation stages (experimental period), it was reduced to 3.1 μm (sd = ±1.5), which was significant (*p* < 0.001). The results from previous studies confirm this observation, stating that secretion of large MFG during early lactation is due to the inability to form sufficient membrane to surround the MFG due to the energy deficit of dairy animals, whereby a high fat concentration acts as a limiting factor of MFGM synthesis [11,49].

The milk fat content throughout lactation was significantly reduced apart from the elevated values on the 156th day (*p* ≤ 0.01). Similarly, milk yield was affected significantly by the lactation period (*p* ≤ 0.001), reaching the highest values in early lactation and the lowest in late lactation, as also mentioned in previous studies [27,32,54]. Milk fat yield also varied (*p* < 0.001) because of changes in milk yield and fat concentration. Fat content in the current study was decreased according to milk production. This is contrary to previous reports, which indicated that milk fat content is lowest when the milk yield reaches the highest values [8,54]. This negative correlation between milk fat content and milk yield has been attributed to the dilution effect when dairy animals produce large amounts of milk. The contrary results in our study may be linked to feeding and the efficiency with which the dietary fat is transferred to the milk [24,55,56]. Furthermore, a study concerning dairy sheep [56], reported that the reason for reduced fat concentration at the end of lactation is the recovery of the animal’s body condition at this stage, which results in the deposition of dietary fat in the adipose tissue.

A significant decrease of some short-chain (C6:0, C8:0) and medium-chain fatty acids (C10:0, C12:0) is observed throughout lactation during the experimental period (*p* ≤ 0.001). The effect of the lactation stage on these FA seems to be the same, with the exception of caproic acid (C6:0), which showed variation only during the experimental period. Myristic (C14:0) and palmitic (C16:0) acids showed a significant difference (*p* ≤ 0.05) among lactation stages, with the highest values on the 121st day. The palmitoleic (C16:1), stearic (C18:0), and oleic (C18:1n9) fatty acids showed the most significant increase from the 156th day of lactation (*p* ≤ 0.001). The increase in stearic (C18:0) content indicates extensive bio-hydrogenation of unsaturated fatty acids in the rumen. Moreover, stearic acid (C18:0) is a precursor for the endogenous mammary synthesis of oleic acid (C18:1n9) [6,16]. On the other hand, a significant reduction is observed in the concentration of linoleic (C18:2cis-6) and a-linolenic (C18:3n3) acid at mid-lactation (121st day) (*p* ≤ 0.05), while this observation was also found for the palmitic (C16:0) and arachidic (C20:0) acids at late lactation. Moreover, the isomers of CLA (C18:2cis-9trans-11, C18:2trans-9trans-11) showed no significant variation throughout lactation. It has previously been reported that high uptake of LCFA prevents de novo synthesis of SCFA [7,46]. However, it has also been pointed out that the mobilization of adipose tissue, which occurs immediately after parturition due to negative energy balance, enhances the incorporation of dietary preformed FA (long-chain fatty acids, especially stearic and oleic acid) [7,46]. As lactation is progresses, the energy balance becomes positive, resulting in an increase of de novo synthesized FA (short- and medium-chain fatty acids). Our results are contrary to the above reports but are consistent with previous studies in sheep [44] and goats [54], also reporting a high content of de novo synthesized FA at early lactation. Regarding cow milk, where a similar FA profile was observed throughout lactation, it was stated that high concentrations of de novo synthesized FA in the early lactation could be attributed to the chemical composition of feed [57]. Concentrated feeding supports the synthesis of short- and medium-chain fatty acids. In addition, this difference in FA pattern may be due to effects on the mammary metabolism of the energy balance, which vary among species and breeds [10]. In conclusion, higher concentrations of short- and medium-chain fatty acids at early lactation indicate reduced delivery of long-chain FA to the mammary gland. In later lactation, the proportions of long-chain FA in milk fat increased significantly. In contrast, significant decreases in C6:0, C8:0, C10:0, C12:0, and C14:0 occurred, indicating less de novo synthesis by the mammary gland. Concerning the amount of SFA, significant variations (*p* ≤ 0.01) were observed during lactation stages. Reduced values at late lactation were found, while higher amounts were found at early and mid-lactation, specifically on the 101st and 121st days of lactation. Opposite results were observed for the MUFA, where a significant increase was shown at early (93rd day) and late lactation (*p* ≤ 0.01). This finding is in accordance with previous studies and could be considered very important since the intake of MUFA by humans contributes positively to the concentration of high-density lipoproteins (HDL), resulting in a reduction of total/HDL cholesterol [8,25,44]. However, recent studies suggested that not all SFA are associated with negative effects on human health. It seems that only the lauric (C12:0), myristic (C14:0), and palmitic (C16:0) acids increase the concentration of total and low-density lipoprotein (LDL) plasma cholesterol in blood [8,25,58]. In contrast, short-chain fatty acids and stearic acid (C18:0) seem to reduce LDL cholesterol. Considering all the above results, late lactation (188th d) showed significant reductions in milk and fat yields, SFA, but also in essential FA (C18:3n3). However, α-linolenic acid (C18:3n3), milk yield, and fat content showed an increase on the 156th day compared to mid- and latest lactation stages. This observation suggests that maybe the lactation period is too long and should have been stopped on the 156th d, to prevent losses in desirable FA. Additionally, high contents of MUFA were found at later lactation stages. Stearic (C18:0) and oleic (C18:1n9) acids, which are related to beneficial effects on health, also showed significant increases in late lactation [8]. These findings highlight that the milk from late lactation, on the 156th day, had a more favorable FA profile for human health compared to early and mid-lactation stages since their FA composition is characterized by higher amounts of MUFA and stearic acid, and a low content of lauric (C12:0) and myristic (C14:0) acids. Our results show a clear effect of the lactation stage on FA profile of sheep milk. Moreover, the given measurements showed a reduction in the average diameter of MFG throughout lactation, which was relative to the reduction of fat concentration. At this point, a significant body of evidence suggests that (i) the ratio of MUFA/SFA is higher in smaller MFG than in larger MFG and (ii) the fat content is positively associated with the size of MFG. These findings agree with other reports in sheep’s [26] and cow’s milk fat [13].

### 3.5. Correlations between the Size and Lipid Composition of the Milk Fat Globules

Significant partial correlations between the average size of MFG and milk FA during lactation are shown in Table 4. At early lactation (93rd d), the average diameter of MFG correlated positively with the concentration of palmitic (C16:0), margaric (C17:0), stearic (C18:0), and arachidonic (C20:4n6) acids and negatively with oleic (C18:1cis-9) and elaidic (C18:1trans-9) acids. Regarding the most abundant FA, it is observed that stearic acid showed a constant positive correlation with the average diameter of MFG throughout lactation. However, correlations concerning palmitic acid and oleic acid have had a different turn. The palmitic acid showed a positive correlation on the 93rd day of lactation and changed to a negative correlation on the 121st day of lactation, while the oleic acid showed negative correlations on the 93rd day and became positive on the 121st day of lactation. In addition, several negative correlations were observed on the 121st day of lactation between the average size of MFG and (C18:2trans-11,cis-15), linoleic (C18:2cis-6), γ-linolenic (C18:3n6), and linolenic (C18:3) acids. In contrast, tridecylic (C13:0) and myristoleic (C14:1) acids showed positive correlations on the 108 day of lactation. In late lactation, a negative correlation was found between the average diameter of MFG and C18:2cis-11,trans-15 and C18:2trans-6 FA.

Our results indicate that the size of MFG is affected by the preformed dietary FA (≥C17:0), with the exception of the associations found for concentrations of tridecylic (C13:0), myristoleic (C14:1), and palmitic (C16:0) acids. Palmitic acid originates from two sources. It is synthesized de novo in the mammary gland, but a portion of this FA is also taken up from the diet. Our findings are consistent with a study [49] where it was observed that (i) palmitic and stearic acids were positively correlated with the average size of MFG and (ii) de novo synthesis of FA did relate with changes in the size of MFG. However, in our study, the concentration of palmitic acid showed that it is related with increases in the average size of MFG in early lactation and then showed a negative correlation in later lactation. Most correlations that were found between the average size of MFG and unsaturated FA with a carbon number ≥C18, were negative, indicating that the high amount of UFA observed in smaller MFG is the consequence of their high membrane/core ratio. Significant correlations also existed between the size of MFG and FA classes (SFA, MUFA, PUFA). The average size of MFG was positively correlated with SFA at early and late lactation. These associations are probably due to an increase in FA supplied by the core at these stages. Oppositely, MUFA at early-lactation and PUFA at mid-lactation showed negative correlations. These results, in agreement with the foregoing observations, highlight the high ratio of UFA/SFA in small MFG.

Concerning the milk fat content, a positive correlation was found with the average size of MFG during lactation. This finding indicates that milk fat content is related to MFG size as well as their FA composition, as mentioned above.

## 4. Conclusions

The relationship between FA composition and the size traits of MFG in sheep milk, under the influence of milking frequency, breed, and lactation stage, were studied. The average size of MFG ranged from 2.8 to 4.5 μm and showed variation between breeds. The high fat content of Karagouniko milk induced the secretion of large globules, resulting in a high concentration of short-chain fatty acids compared to Chios milk with low fat content. However, milk FA profile and size of MFG were affected to a higher extent by the lactation stage rather than by the breed. The relative proportions of most short- and medium-chain FA decreased as lactation progressed, whereas MUFA proportion increased. Therefore, the increased secretion of small MFG at advanced lactation stages demonstrated the high ratio of MUFA/SFA in small MFG, contrary to large fat globules. Milking frequency did not cause significant changes in FA profile and size of MFG. However, shorter milking intervals should be investigated. Moreover, significant associations among long-chain fatty acids (≥C16:0) and MFG size were found during lactation. Therefore, there is a possibility to regulate the quality of sheep milk and its products. The results of our study seem to hold promise for future research aimed at improving the effects of various factors (genetic, physiological, nutritional) to increase concentrations of fatty acids in milk that are beneficial for human health without degrading the sensory quality of dairy products.

## Figures and Tables

**Table 1:** Main effects of milking frequency (once (1×) vs. twice (2×) daily) on daily milk yield (mL/d), daily fat yield (g/d) and fat content (%), and on sheep’s MFG size parameters and fatty acids (%total FAMEs) (mean values ± S.E.M) during the experimental period.

Traits	1× Milking	2× Milking	*p* ^1^
Milk yield (mL/d)	785.21 ± 93.91	998.25 ± 101.82	ns
Fat yield (g/d)	38.89 ± 4.21	45.73 ± 4.57	ns
Fat %	4.97 ± 0.22	4.95 ± 0.24	ns
D_(4,3)_	3.06 ± 0.19	3.20 ± 0.20	ns
D_(3,2)_	0.20 ± 0.01	0.21 ± 0.01	ns
d_(0.1)_	0.08 ± 0.00	0.08 ± 0.00	ns
d_(0.5)_	0.58 ± 0.14	0.81 ± 0.15	ns
d_(0.9)_	7.64 ± 0.45	7.92 ± 0.49	ns
C4:0	1.22 ± 0.03	1.21 ± 0.03	ns
C6:0	0.83 ± 0.03	0.80 ± 0.03	ns
C8:0	0.86 ± 0.04	0.79 ± 0.04	ns
C10:0	3.02 ± 0.14	2.75 ± 0.15	ns
C11:0	0.12 ± 0.03	0.10 ± 0.03	ns
C12:0	2.54 ± 0.10	2.31 ± 0.11	ns
C13:0	0.14 ± 0.01	0.13 ± 0.01	ns
C14:0	10.18 ± 0.24	9.98 ± 0.26	ns
anteiso-C15:0	0.27 ± 0.01	0.26 ± 0.01	ns
C14:1	0.45 ± 0.01	0.43 ± 0.01	ns
C15:0	1.03 ± 0.02	0.98 ± 0.03	ns
iso-16:0	0.34 ± 0.01	0.32 ± 0.01	ns
C16:0	30.50 ± 0.43	30.66 ± 0.46	ns
iso-C17:0	0.34 ± 0.03	0.31 ± 0.03	ns
anteiso-C17:0	0.54 ± 0.02	0.55 ± 0.02	ns
C16:1	1.41 ± 0.06	1.37 ± 0.07	ns
C17:0	0.78 ± 0.04	0.78 ± 0.04	ns
C17:1	0.28 ± 0.02	0.26 ± 0.02	ns
C18:0	12.43 ± 0.63	12.67 ± 0.69	ns
C18:1trans-9	1.09 ± 0.43	2.06 ± 0.47	ns
C18:1cis-9	21.05 ± 0.57	20.87 ± 0.62	ns
C18:1 cis-11, trans-15	0.36 ± 0.02	0.34 ± 0.02	ns
C18:1cis-12	0.24 ± 0.01	0.25 ± 0.01	ns
C18:1cis-13	0.19 ± 0.01	0.19 ± 0.01	ns
C18:2trans-6	0.54 ± 0.02	0.48 ± 0.02	**
C18:2trans-8, cis-13	0.46 ± 0.02	0.41 ± 0.02	*
C18:2trans-11, cis-15	0.34 ± 0.01	0.30 ± 0.02	ns
C18:2cis-6	3.87 ± 0.15	3.93 ± 0.16	ns
C20:0	0.61 ± 0.02	0.65 ± 0.02	ns
C20:1n9	0.10 ± 0.02	0.09 ± 0.03	ns
C18:3n3	0.27 ± 0.01	0.28 ± 0.01	ns
C18:2 cis-9, trans-11	1.85 ± 0.12	1.82 ± 0.13	ns
C18:2trans-10, cis-12	0.10 ± 0.03	0.18 ± 0.05	ns
C18:2trans-9, trans-11	0.32 ± 0.05	0.34 ± 0.06	ns
C22:0	0.24 ± 0.01	0.25 ± 0.01	ns
C20:4n6	0.26 ± 0.01	0.24 ± 0.01	ns
C23:0	0.08 ± 0.01	0.09 ± 0.01	ns
NIFA	0.71 ± 0.03	0.64 ± 0.031	ns
SFA	66.00 ± 0.82	65.52 ± 0.88	ns
MUFA	25.12 ± 0.80	25.82 ± 0.86	ns
PUFA	8.09 ± 0.22	7.92 ± 0.24	ns
AI	2.28 ± 0.08	2.21 ± 0.09	ns
TI	3.46 ± 0.14	3.40 ± 0.15	ns

^1^ *: Significant differences at *p* ≤ 0.05; **: Significant differences at *p* ≤ 0.01; ns: Non-significant differences. NIFA: Non-identified fatty acids; SFA: Saturated fatty acids; MUFA: Mono-unsaturated fatty acids: PUFA: Poly-unsaturated fatty acids. AI: Atherogenicity Index; TI: Thrombogenicity Index.

**Table 2:** Main effects of breed on daily milk yield (mL/d), daily fat yield (g/d), and fat content (%) and on sheep’s MFG size parameters and fatty acids (% total FAMEs) (mean values ± S.E.M), during the preliminary and experimental periods.

Traits	Preliminary Period (Twice Daily Milking)			Experimental Period (Once and Twice Daily Milking)		
	Karagouniko	Chios	*p* ^1^	Karagouniko	Chios	*p* ^1^
Milk yield (mL/d)	847.08 ± 100.70	1453.13 ± 123.33	***	658.96 ± 96.74	1124.50 ± 99.13	*
Fat (g/d)	53.50 ± 7.10	78.62 ± 7.94	*	33.66 ± 4.34	50.96 ± 4.44	**
Fat %	6.15 ± 0.33	5.53 ± 0.37	ns	5.17 ± 0.23	4.75 ± 0.23	ns
D_(4,3)_	4.57 ± 0.37	3.92 ± 0.45	ns	3.39 ± 0.19	2.86 ± 0.20	ns
D_(3,2)_	0.26 ± 0.12	0.24 ± 0.15	ns	0.20 ± 0.01	0.21 ± 0.01	ns
d_(0.1)_	0.09 ± 0.00	0.09 ± 0.00	ns	0.08 ± 0.00	0.08 ± 0.00	ns
d_(0.5)_	2.13 ± 0.38	1.93 ± 0.46	ns	0.48 ± 0.14	0.91 ± 0.14	*
d_(0.9)_	10.96 ± 0.83	9.49 ± 1.02	ns	8.46 ± 0.46	7.11 ± 0.48	*
C4:0	1.27 ± 0.03	1.13 ± 0.04	*	1.26 ± 0.03	1.16 ± 0.03	**
C6:0	1.46 ± 0.26	0.92 ± 0.32	ns	0.89 ± 0.03	0.75 ± 0.03	***
C8:0	1.26 ± 0.08	0.98 ± 0.10	*	0.93 ± 0.04	0.72 ± 0.04	***
C10:0	4.32 ± 0.30	3.51 ± 0.37	ns	3.23 ± 0.14	2.53 ± 0.15	**
C11:0	0.13 ± 0.01	0.09 ± 0.01	*	0.12 ± 0.02	0.09 ± 0.04	ns
C12:0	3.12 ± 0.18	2.68 ± 0.23	ns	2.61 ± 0.10	2.24 ± 0.11	*
C13:0	0.12 ± 0.01	0.12 ± 0.01	ns	0.15 ± 0.01	0.12 ± 0.01	ns
C14:0	10.22 ± 0.22	10.53 ± 0.27	ns	9.96 ± 0.25	10.19 ± 0.25	ns
anteisoC15:0	0.25 ± 0.02	0.24 ± 0.02	ns	0.27 ± 0.01	0.26 ± 0.01	ns
C14:1	0.42 ± 0.01	0.40 ± 0.02	ns	0.45 ± 0.01	0.43 ± 0.01	ns
C15:0	1.06 ± 0.03	1.10 ± 0.04	ns	1.02 ± 0.02	0.99 ± 0.03	ns
iso-16:0	0.33 ± 0.01	0.33 ± 0.01	ns	0.33 ± 0.01	0.33 ± 0.01	ns
C16:0	28.98 ± 0.64	31.27 ± 0.79	*	29.20 ± 0.44	31.97 ± 0.45	***
iso-C17:0	0.30 ± 0.04	0.34 ± 0.05	ns	0.33 ± 0.03	0.32 ± 0.03	ns
anteisoC17:0	0.43 ± 0.03	0.44 ± 0.04	ns	0.55 ± 0.02	0.53 ± 0.02	ns
C16:1	1.22 ± 0.08	1.28 ± 0.10	ns	1.42 ± 0.07	1.36 ± 0.07	ns
C17:0	0.74 ± 0.03	0.81 ± 0.03	ns	0.79 ± 0.04	0.77 ± 0.04	ns
C17:1	0.28 ± 0.03	0.31 ± 0.04	ns	0.27 ± 0.02	0.27 ± 0.02	ns
C18:0	11.81 ± 0.88	11.17 ± 1.08	ns	13.28 ± 0.65	11.82 ± 0.67	ns
C18:1trans-9	2.90 ± 0.90	2.85 ± 1.10	ns	1.64 ± 0.45	1.51 ± 0.46	ns
C18:1cis-9	19.10 ± 0.72	18.68 ± 0.88	ns	21.07 ± 0.59	20.84 ± 0.60	ns
C18:1 cis-11, trans-15	0.30 ± 0.03	0.34 ± 0.03	ns	0.32 ± 0.02	0.38 ± 0.02	ns
C18:1cis-12	0.26 ± 0.02	0.21 ± 0.02	ns	0.25 ± 0,01	0.25 ± 0.01	ns
C18:1cis-13	0.19 ± 0.01	0.19 ± 0.01	ns	0.19 ± 0,01	0.19 ± 0.01	ns
C18:2trans-6	0.44 ± 0.02	0.44 ± 0.03	ns	0.51 ± 0.02	0.52 ± 0.02	ns
C18:2trans-8, cis-13	0.39 ± 0.02	0.36 ± 0.03	ns	0.42 ± 0.02	0.44 ± 0.02	ns
C18:2trans-11, cis-15	0.28 ± 0.02	0.35 ± 0.02	*	0.31 ± 0.02	0.33 ± 0.02	ns
C18:2cis-6	3.79 ± 0.22	4.26 ± 0.27	ns	3.77 ± 0,15	4.03 ± 0.15	ns
C20:0	0.50 ± 0.02	0.49 ± 0,02	ns	0.63 ± 0.02	0.62 ± 0.02	ns
C20:1n9	0.09 ± 0.02	0.07 ± 0.02	ns	0.12 ± 0.03	0.07 ± 0.03	ns
C18:3n3	0.36 ± 0.02	0.41 ± 0.02	ns	0.28 ± 0.01	0.28 ± 0.01	ns
C18:2 cis-9, trans-11	1.73 ± 0.20	1.87 ± 0.24	ns	1.79 ± 0.01	1.87 ± 0.12	ns
C18:2trans-10, cis-12	0.13 ± 0.02	0.15 ± 0.02	ns	0.13 ± 0.03	0.15 ± 0.05	ns
C18:2trans-9, trans-11	0.29 ± 0.09	0.26 ± 0.11	ns	0.29 ± 0.05	0.37 ± 0.06	ns
C22:0	0.22 ± 0.01	0.20 ± 0.02	ns	0.25 ± 0.01	0.23 ± 0.01	ns
C20:4n6	0.25 ± 0.02	0.27 ± 0.02	ns	0.25 ± 0.01	0.25 ± 0.01	ns
C23:0	0.09 ± 0.01	0.08 ± 0.01	ns	0.09 ± 0.01	0.07 ± 0.01	ns
NIFA	0.86 ± 0.05	0.83 ± 0.06	ns	0.66 ± 0.03	0.69 ± 0.03	ns
SFA	66.61 ± 1.30	66.42 ± 1.60	ns	65.84 ± 0.84	65.67 ± 0.86	ns
MUFA	24.75 ± 1.18	24.37 ± 1.45	ns	25.66 ± 0.82	25.28 ± 0.84	ns
PUFA	7.72 ± 0.25	8.28 ± 0.31	ns	7.78 ± 0.23	8.23 ± 0.23	ns
AI	2.32 ± 0.11	2.40 ± 0.14	ns	2.20 ± 0.08	2.29 ± 0.08	ns
TI	3.37 ± 0.19	3.46 ± 0.24	ns	3.39 ± 0.14	3.46 ± 0.15	ns

^1^ *: Significant differences at *p* ≤ 0.05; **: Significant differences at *p* ≤ 0.01; ***: Significant differences at *p* ≤ 0.001; ns: Non-significant differences. NIFA: Non-identified fatty acids; SFA: Saturated fatty acids; MUFA: Mono-unsaturated fatty acids: PUFA: Poly-unsaturated fatty acids; AI: Atherogenicity Index; TI: Thrombogenicity Index.

**Table 3:** Main effects of lactation stage on sheep’s daily milk yield (mL), daily fat yield (g), and fat content (%), and on sheep’s MFG size parameters and fatty acids (% total FAMEs) (mean values ± S.E.M).

traits	Preliminary Period (Twice Daily Milking)			Experimental Period (Once and Twice Daily Milking)				
	93rd d	101st d	*p* ^1^	108th d	121st d	156th d	188th d	*p* ^1^
Milk yield (mL/d)	1168.54 ± 88.95	1131.67 ± 77.21	ns	927.81 ^ab^ ± 84.80	903.12 ^abc^ ± 70.06	974.05 ^ab^ ± 76.33	761.94 ^c^ ± 75.47	***
Fat (g/d)	67.64 ± 5.84	64.66 ± 5.53	ns	-	43.12 ^a^ ± 2.75	50.04 ^a^ ± 4.08	33.77 ^b^ ± 3.37	***
Fat %	5.87 ± 0.29	5.81 ± 0.27	ns	-	5.01 ^ab^ ± 0.17	5.23 ^a^ ± 0.15	4.65 ^bc^ ± 0.24	**
D_(4,3)_	4.04 ± 0.34	4.45 ± 0.387	ns	3.32 ± 0.29	2.81 ± 0.26	3.25 ± 0.28	3.13 ± 0.36	ns
D_(3,2)_	0.20 ± 0.01	0.21 ± 0.01	ns	0.21 ± 0.01	0.20 ± 0.01	0.20 ± 0.01	0.22 ± 0.01	ns
d_(0.1)_	0.09 ± 0.002	0.09 ± 0.002	ns	0.08 ± 0.001	0.08 ± 0.001	0.08 ± 0.001	0.09 ± 0.001	ns
d_(0.5)_	1.73 ± 0.36	2.32 ± 0.32	ns	0.70 ± 0.16	0.50 ± 0.136	0.80 ± 0.18	0.78 ± 0.22	ns
d_(0.9)_	9.74 ± 0.66	10.71 ± 0.98	ns	8.37 ± 0.63	6.87 ± 0.38	8.24 ± 0.57	7.64 ± 0.73	ns
C4:0	1.23 ± 0.03	1.17 ± 0.03	ns	1.21 ± 0.03	1.26 ± 0.03	1.20 ± 0.03	1.19 ± 0.03	ns
C6:0	1.11 ± 0.05	1.27 ± 0.41	ns	0.89 ^a^ ± 0.03	0.85 ^a^ ± 0.04	0.82 ^a^ ± 0.03	0.71 ^b^ ± 0.03	**
C8:0	1.25 ± 0.08	0.98 ± 0.05	***	0.92 ^a^ ± 0.04	0.91 ^a^ ± 0.05	0.80 ^a^ ± 0.03	0.67 ^b^ ± 0.04	***
C10:0	4.34 ± 0.31	3.49 ± 0.20	***	3.16 ^a^ ± 0.11	3.29 ^a^ ± 0.15	2.74 ^b^ ± 0.13	2.35 ^c^ ± 0.15	***
C11:0	0.12 ± 0.01	0.10 ± 0.01	**	0.11 ± 0.02	0.12 ± 0.03	0.11 ± 0.03	0.08 ± 0.02	ns
C12:0	3.14 ± 0.10	2.66 ± 0.12	***	2.57 ^a^ ± 0.07	2.66 ^a^ ± 0.10	2.25 ^b^ ± 0.07	2.22 ^b^ ± 0.12	***
C13:0	0.10 ± 0.01	0.13 ± 0.01	**	0.12 ± 0.01	0.14 ± 0.02	0.15 ± 0.02	0.13 ± 0.01	ns
C14:0	10.33 ± 0.30	10.41 ± 0.20	ns	10.15 ^b^ ± 0.19	10.87 ^a^ ± 0.24	9.70 ^b^ ± 0.23	9.59 ^b^ ± 0.24	**
anteisoC15:0	0.23 ± 0.02	0.25 ± 0.02	ns	0.25 ^b^ ± 0.01	0.26 ^ab^ ± 0.01	0.29 ^a^ ± 0.01	0.26 ^ab^ ± 0.01	**
C14:1	0.37 ± 0.02	0.44 ± 0.01	*	0.41 ^b^ ± 0.01	0.45 ^ab^ ± 0.04	0.47 ^a^ ± 0.01	0.43 ^ab^ ± 0.01	*
C15:0	1.05 ± 0.03	1.11 ± 0.04	ns	0.97 ± 0.03	1.01 ± 0.03	1.06 ± 0.02	0.99 ± 0.03	ns
iso-16:0	0.31 ± 0.01	0.32 ± 0.01	ns	0.34 ± 0.02	0.34 ± 0.01	0.31 ± 0.01	0.35 ± 0.02	ns
C16:0	29.16 ± 0.75	31.09 ± 0.46	*	30.44 ^b^ ± 0.45	32.60 ^a^ ± 0.59	29.43 ^b^ ± 0.46	29.85 ^b^ ± 0.43	*
iso-C17:0	0.28 ± 0.05	0.37 ± 0.02	*	0.28 ± 0.03	0.33 ± 0.03	0.30 ± 0.03	0.39 ± 0.04	ns
anteisoC17:0	0.31 ± 0.04	0.56 ± 0.03	***	0.45 ^b^ ± 0.03	0.65 ^a^ ± 0.05	0.56 ^a^ ± 0.03	0.49 ^ab^ ± 0.04	**
C16:1	0.90 ± 0.10	1.60 ± 0.05	***	1.45 ^a^ ± 0.07	1.01 ^b^ ± 0.08	1.47 ^a^ ± 0.09	1.62 ^a^ ± 0.04	***
C17:0	0.70 ± 0.04	0.85 ± 0.03	*	0.73 ^b^ ± 0.03	0.62 ^c^ ± 0.03	0.99 ^a^ ± 0.07	0.79 ^b^ ± 0.03	***
C17:1	0.33 ± 0.05	0.26 ± 0.01	ns	0.28 ± 0.01	0.25 ± 0.01	0.30 ± 0.04	0.25 ± 0.02	ns
C18:0	10.14 ± 0.86	12.84 ± 0.65	***	12.43 ^ab^ ± 0.60	11.52 ^b^ ± 0.49	13.23 ^a^ ± 0.43	13.03 ^a^ ± 0.71	***
C18:1trans-9	3.85 ± 0.87	1.90 ± 0.72	*	1.54 ± 0.53	1.85 ± 0.39	0.78 ± 0.28	2.14 ± 0.67	ns
C18:1cis-9	20.22 ± 0.65	17.56 ± 0.73	**	20.08 ^abc^ ± 0.72	18.98 ^c^ ± 0.69	22.50 ^a^ ± 0.65	22.26 ^ad^ ± 0.74	***
C18:1 cis-11, trans-15	0.39 ± 0.04	0.25 ± 0.02	*	0.35 ^a^ ± 0.02	0.45 ^ab^ ± 0.05	0.27 ^ac^ ± 0.03	0.33 ^a^ ± 0.03	*
C18:1cis-12	0.25 ± 0.03	0.26 ± 0.02	ns	0.20 ^a^ ± 0.01	0.26 ^ab^ ± 0.03	0.25 ^ab^ ± 0.02	0.27 ± 0.01	***
C18:1cis-13	0.19 ± 0.01	0.20 ± 0.03	ns	0.19 ± 0.01	0.20 ± 0.03	0.18 ± 0.02	0.19 ± 0.01	ns
C18:2trans-6	0.42 ± 0.02	0.46 ± 0.02	ns	0.54 ^a^ ± 0.02	0.47 ^b^ ± 0.02	0.48 ^b^ ± 0.02	0.56 ^a^ ± 0.02	***
C18:2trans-8, cis-13	0.33 ± 0.02	0.43 ± 0.02	***	0.48 ^a^ ± 0.02	0.35 ^b^ ± 0.02	0.41 ^b^ ± 0.02	0.50 ^a^ ± 0.02	***
C18:2trans-11, cis-15	0.26 ± 0.03	0.37 ± 0.03	ns	0.32 ± 0.01	0.32 ± 0.03	0.33 ± 0.02	0.32 ± 0.03	ns
C18:2cis-6	3.97 ± 0.30	4.08 ± 0.12	ns	4.27 ^a^ ± 0.08	3.97 ^ab^ ± 0.11	3.71 ^ab^ ± 0.28	3.66 ^b^ ± 0.18	*
C20:0	0.41 ± 0.02	0.58 ± 0.02	***	0.72 ^a^ ± 0.02	0.63 ^b^ ± 0.03	0.57 ^b^ ± 0.02	0.58 ^b^ ± 0.03	***
C20:1n9	0.06 ± 0.03	0.09 ± 0.01	ns	0.09 ± 0.02	0.08 ± 0.03	0.07 ± 0.01	0.14 ± 0.02	ns
C18:3n3	0.40 ± 0.02	0.37 ± 0.02	ns	0.30 ^a^ ± 0.01	0.28 ^ab^ ± 0.03	0.31 ^a^ ± 0.01	0.22 ^b^ ± 0.01	***
C18:2 cis-9, trans-11	1.89 ± 0.26	1.71 ± 0.08	ns	1.75 ± 0.08	1.68 ± 0.10	2.10 ± 0.17	1.80 ± 0.10	ns
C18:2trans-10, cis-12	0.14 ± 0.02	0.15 ± 0.02	ns	0.14 ± 0.03	0.20 ± 0.04	0.10 ± 0.01	0.12 ± 0.04	ns
C18:2trans-9, trans-11	0.40 ± 0.14	0.15 ± 0.01	ns	0.29 ± 0.02	0.31 ± 0.02	0.42 ± 0.14	0.29 ± 0.04	ns
C22:0	0.19 ± 0.02	0.23 ± 0.01	ns	0.25 ± 0.01	0.24 ± 0.01	0.23 ± 0.01	0.23 ± 0.01	ns
C20:4n6	0.25 ± 0.01	0.27 ± 0.02	ns	0.25 ± 0.01	0.25 ± 0.01	0.25 ± 0.01	0.24 ± 0.01	ns
C23:0	0.09 ± 0.02	0.08 ± 0.01	ns	0.07 ± 0.01	0.07 ± 0.01	0.11 ± 0.02	0.07 ± 0.01	ns
NIFA	0.82 ± 0.06	0.87 ± 0.06	ns	0.75 ^a^ ± 0.04	0.68 ^ab^ ± 0.05	0.72 ^ab^ ± 0.04	0.55 ^b^ ± 0.05	*
SFA	64.49 ± 1.24	68.54 ± 1.07	***	66.13 ^cd^ ± 0.80	68.14 ^ac^ ± 0.88	64.77 ^bd^ ± 0.75	63.99 ^d^ ± 0.88	**
MUFA	26.57 ± 1.13	22.55 ± 1.08	**	24.61 ^bcd^ ± 0.76	23.45 ^b^ ± 0.87	26.20 ^acd^ ± 0.80	27.61 ^a^ ± 0.94	**
PUFA	8.00 ± 0.29	8.00 ± 0.18	ns	8.46 ^a^ ± 0.14	7.69 ^b^ ± 0.20	8.08 ^ab^ ± 0.24	7.79 ^ab^ ± 0.25	**
AI	2.19 ± 0.11	2.53 ± 0.11	**	2.27 ^b^ ± 0.08	2.60 ^a^ ± 0.11	2.09 ^b^ ± 0.08	2.03 ^bc^ ± 0.07	***
TI	3.08 ± 0.18	3.75 ± 0.17	**	3.42 ^ac^ ± 0.13	3.8 ^a^ ± 0.17	3.27 ^bc^ ± 0.11	3.21 ^bc^ ± 0.13	**

^a,b,c,d^: Different superscript letters within the same row indicate statistically significant differences ^1^ *: at *p* ≤ 0.05; **: at *p* ≤ 0.01; ***: at *p* ≤ 0.001; ns: Non-significant differences; -: Missing data. NIFA: Non-identified fatty acids; SFA: Saturated fatty acids; MUFA: Mono-unsaturated fatty acids: PUFA: Poly-unsaturated fatty acids; AI: Atherogenicity Index; TI: Thrombogenicity Index.

**Table 4:** Partial correlations among the size of MFG and FA throughout lactation.

Traits	D_[4, 3]_ (Average Diameter)					
	93rd	101st	108th	121st	156th	188th
Milk yield (mL/d)	ns	ns	ns	ns	ns	−0.398 *
Fat yield (g/d)	ns	ns	ns	ns	ns	ns
Fat%	0.463 *	0.695 **	ns	ns	0.530 **	0.594 **
C4:0	ns	ns	ns	ns	ns	ns
C6:0	ns	ns	ns	ns	ns	ns
C8:0	ns	ns	ns	ns	ns	ns
C10:0	ns	ns	ns	ns	ns	ns
C12:0	ns	ns	ns	ns	ns	ns
C13:0	ns	ns	0.491 **	ns	ns	ns
C14:0	ns	ns	ns	ns	ns	ns
C14:1	ns	ns	0.390 *	ns	ns	ns
C15:0	ns	ns	ns	ns	ns	ns
C16:0	0.480 *	ns	ns	−0.399 *	ns	ns
C16:1	ns	ns	ns	ns	ns	ns
C17:0	0.529 *	ns	ns	ns	ns	ns
C17:1	ns	ns	ns	ns	ns	ns
C18:0	0.570 **	ns	0.495 **	ns	ns	0.507 **
C18:1trans-9	−0.621 **	ns	ns	ns	ns	ns
C18:1cis-9	−0.433 *	ns	ns	0.434 *	ns	ns
C18:1cis-11, trans-15	ns	ns	ns	ns	ns	−0.496 **
C18:1cis-12	ns	ns	ns	ns	ns	ns
C18:2trans-6	ns	ns	ns	ns	−0.388 *	ns
C18:2trans-11, cis-15	ns	ns	ns	−0.579 **	ns	ns
C18:2cis-6	ns	ns	ns	−0.492 *	ns	ns
C20:0	ns	ns	ns	ns	ns	ns
C18:3n6	ns	ns	−0.604 **	ns	ns	ns
C18:3	ns	ns	ns	−0.948 *	ns	ns
C20:1n9	ns	ns	ns	ns	ns	ns
C18:3n3	ns	ns	ns	ns	ns	ns
C18:2cis-9, trans-11	ns	ns	ns	ns	ns	ns
C18:2trans-10, cis-12	ns	ns	ns	ns	ns	ns
C18:2trans-9, trans-11	ns	ns	ns	ns	ns	ns
C22:0	ns	ns	ns	ns	ns	ns
C20:4n6	0.630 **	ns	ns	ns	ns	ns
C23:0	ns	ns	ns	ns	ns	ns
SFA	0.717 **	ns	ns	ns	ns	0.458 *
MUFA	−0.746 **	ns	ns	ns	ns	ns
PUFA	ns	ns	ns	−0.415 *	ns	ns

Significant correlations: * *p* ≤ 0.05; ** *p* ≤ 0.01; ns: Non-significant. SFA: Saturated fatty acids; MUFA: Mono-unsaturated fatty acids: PUFA: Poly-unsaturated fatty acids.

## Data Availability

Data generated during the study are available from the corresponding author upon request.

## Supplementary Materials

The following supporting information can be downloaded at: https://www.mdpi.com/article/10.3390/foods12132446/s1, Table S1: Composition of ewes diet, Figure S1: Schematic description of milk samples collection.
