# Supplementary material for: The Effect of Milking Frequency, Breed, and Stage of Lactation on the Milk Fat Globule Size and Fatty Acid Composition in Sheep’s Milk
Source: Foods. 2023 Jun 22;12(13):2446. doi: 10.3390/foods12132446 (PMC10340205; doi:10.3390/foods12132446)
Supplement: Supplementary file 1 [file foods-12-02446-s001.zip › foods-2460356-supplementary.pdf]

Table S1: Composition of ewes ‘diet.

Components (%)	Basal ration	Lactation ration
Maize	74.06	64.41
Sunflower meal	6.99	0.00
Soybean meal	2.72	24.82
Rice bran	5.00	0.28
Calcium phosphate	2.52	1.66
Limestone	1.80	1.63
Molasse	6.00	6.00
Salt	0.50	0.70
Vitamins & Trace elements	0.401	0.502

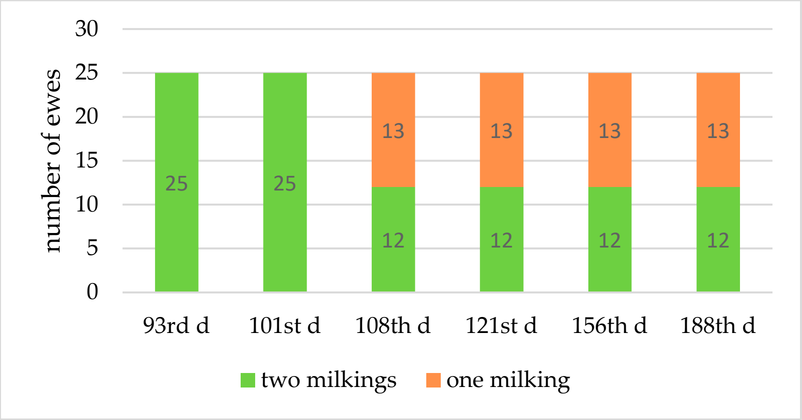

Figure S1: Schematic description of milk samples collection.
